# Exclusive breastfeeding among Indonesian working mothers: does early initiation of breastfeeding matter?

**DOI:** 10.1186/s12889-024-18619-2

**Published:** 2024-05-03

**Authors:** Isyatun Mardhiyah Syahri, Agung Dwi Laksono, Maya Fitria, Nikmatur Rohmah, Masruroh Masruroh, Mara Ipa

**Affiliations:** 1 Faculty of Public Health, Universitas Sumatera Utara, Medan, Indonesia; 2 National Research and Innovation Agency Republic of Indonesia, Jakarta, Indonesia; 3 Faculty of Health Sciences, Muhammadiyah University of Jember, Jember, Indonesia; 4 Faculty of Health Science, Universitas Pesantren Tinggi Darul Ulum, Jombang, Indonesia

**Keywords:** Exclusive breastfeeding, Early initiation of breastfeeding, Working mother, Public health nutrition, Health policy

## Abstract

**Background:**

Early initiation of breastfeeding (EIBF) is a starting point that lays the foundation for breastfeeding and bonding between mother and baby. Meanwhile, working mothers are one of the vulnerable groups for the success of exclusive breastfeeding (EBF). The study analyzed the role of EIBF on EBF among Indonesian working mothers.

**Methods:**

The cross-sectional study examined secondary data from the 2021 Indonesian National Nutritional Status Survey. The study analyzed 4,003 respondents. We examined EBF practice as an outcome variable and EIBF as an exposure variable. We included nine control variables (residence, maternal age, marital, education, prenatal classes, wealth, infant age, sex, and birth weight). All variables were assessed by questionnaire. The study employed a binary logistic regression test in the last stage.

**Results:**

The result showed that the proportion of EBF among working mothers in Indonesia in 2021 was 51.9%. Based on EIBF, Indonesian working mothers with EIBF were 2.053 times more likely than those without to perform EBF (*p* < 0.001; AOR 2.053; 95% CI 2.028–2.077). Moreover, the study also found control variables related to EBF in Indonesia: residence, maternal age, marital, education, prenatal classes, wealth, infant age, sex, and birth weight.

**Conclusion:**

The study concluded that EIBF was related to EBF. Indonesian working mothers with EIBF were two times more likely than those without to perform EBF. The government needs to release policies that strengthen the occurrence of EIBF in working mothers to increase EBF coverage.

## Introduction

 Breastfeeding and human milk are the normative standards for infant feeding and nutrition. WHO recommends mothers worldwide to exclusively breastfeed infants for the child’s first six months to achieve optimal growth, development, and health [1], meaning no other foods or liquids are provided, including water. Infants should be breastfed on demand, as often as the child wants, day and night. The American Academy of Pediatrics also recommends EBF approximately six months after delivery [2]. After that, they should be given nutritious complementary foods and continue breastfeeding for up to two years or beyond. EBF for six months has many benefits for babies and mothers. The most crucial benefit for infants is protection against gastrointestinal infections. The risk of death from diarrhea and other diseases may increase in partially or not breastfed infants. They reduced the risk of illness due to different conditions and minimized the risk of obesity [3, 4].

In some studies, breastfeeding has been linked to higher IQ (Intelligence Quotient) scores in later childhood. Exclusively breastfed girls had higher IQ results than bottle-fed girls [5]. Moreover, physical closeness, skin-to-skin touching, and eye contact help the baby bond with the mother and feel secure. Breast milk’s nutritional content is sufficient for babies’ growth and development needs up to six months. The benefits for the mother are that it can reduce the risk of ovarian and breast cancer and help with the spacing of pregnancies (EBF for babies under six months has a hormonal effect that often causes a lack of menstruation). The condition is a natural birth control method known as the Lactational Amenorrhea Method [3, 4].

In Indonesia, the coverage of mothers giving EBF is 69.7%. The condition has exceeded the national target figure of 45% in the 2020–2021 Ministry of Health performance report [6]. However, it is still considered standard compared to the target of the Ministry of Health’s strategic plan for the 2020–2024 period, which is 69%. Overall, as many as 20 provinces in Indonesia have EBF coverage below the target for the Ministry of Health’s strategic plan [6]. Around 40% of babies are EBF globally, increasing to 50% by 2025. However, this has yet to reach the global target coverage of 100% recommended by UNICEF. According to a study conducted in Ghana, the prevalence of EBF in infants under six months of age peaked in 2008 at 62.8% and dropped to 42.9% in 2017. For the last four surveys, the proportion of infants aged 6 to 11 months receiving age-appropriate breast milk has remained steady, ranging from 79.3% in 2008 to 81.1% in 2017. Breastfeeding among 12- to 23-month-olds that was age-appropriate fell from 77.8% in 2003 to 61.2% in 2017 [7]. In contrast, research in Ethiopia found that 47% of infants perform EBF for the first six months, and 81.8% of the children weaned within an hour of delivery [8].

The success of implementing EBF is influenced by several factors, including the mother’s employment status, knowledge of breastfeeding, delivery method, parity, perceptions of lack of breastfeeding, attitude to breastfeeding, self-efficacy of breastfeeding, and intention [9]. Family sociodemographic, maternal and child factors, misunderstandings about EBF, mother’s education, occupation, antenatal and postnatal attendance, place of delivery, and pressure from mother-in-law and grandmother influenced EBF [10–12]. The critical factors for facilitating breastfeeding are opportunities for close and prolonged physical contact with the baby, positive relationships, support from staff and peers, and facilities for breastfeeding when the baby shows sucking cues [13]. Previous research in Africa confirms that knowledge and attitudes, infrastructure, and supply of health facilities are growing concerns, such as overcrowding and lack of privacy during breastfeeding counseling, which reduces the openness and comfort of mothers, especially HIV-positive [14]. For factors determining the success of EBF, a study emphasizes the bond and interaction between mother and baby, and the method of breastfeeding is a determining factor in EBF [15].

Much research reported the factors that influence the success of EBF, but very little is related to working mothers. The number of female workers is equivalent to 38.98% of the total workers in Indonesia. In the Indonesian context, the condition of working mothers is an obstacle to breastfeeding because of their multiple roles; besides earning a living, they also do household chores. Household pressure forces mothers to work, thus changing the practice of breastfeeding and adding breast milk substitutes, one of the factors being less than three months of maternity leave [16]. Some mothers express breast milk at work, but sociocultural challenges affect the practice of breastfeeding, such as women who are not good at breastfeeding in public places, which contributes to reduced respect for women who breastfeed at the workplace [17]. Another factor, according to a previous study, is that working mothers cannot bring their children to work because their work schedule is not conducive. They do not have enough time at work, so they are forced to provide food for their children because breastfeeding alone is not enough [18]. One of the findings is the condition of going to work very early and coming home late at night, not being able to breastfeed the baby consistently, and the discomfort of expressing milk for the baby because they believe the babysitter will not handle it hygienically [19]. Rather than entrusting it to a babysitter who is an outsider, mothers feel more helped by family support [20].

Moreover, the challenge of realizing EIBF is not easy, primarily related to the choice of place of birth. Several studies have found that the success of EIBF is often associated with the choice of place of birth, including the type of childbirth [21, 22]. The above conditions still indicate success factors for giving EBF to working mothers but have not been associated with EIBF. Regarding the background narration, the study analyzed the role of EIBF on EBF among Indonesian working mothers. It is crucial to provide solutions and design policies for the government and company leaders to increase the achievement of EBF for working mothers.

## Materials and methods

### Study design and data source

The study examined secondary data from the Indonesian National Nutritional Status Survey conducted in 2021. The Indonesian Ministry of Health conducted the cross-sectional survey on a national level. The poll was designed with stratified two-stage sampling from January-December 2021. The survey collected 152,228 households.

The study included all Indonesian working mothers with infants under six months (1–5 months) population. The study examined 4,003 Indonesian working mothers.

### Outcome variable

The study employed EBF practices of mothers of infants less than six months as an outcome variable. The EBF comprises No and Yes. Mothers who reported that they had fed their infants any food other than breast milk received a “No” rating; those who said they had not were given a “Yes” rating [23].

### Exposure variables

The study used EIBF as an exposure variable. The research defined EIBF as the start of mothers’ nursing neonates during the first hour of birth to ensure that the infant receives colostrum [24]. The study split EIBF into No and Yes.

### Control variables

The study examines nine control variables: residence type, maternal age group, maternal marital status, education level, prenatal classes, wealth status, infant age, sex of infant, and birth weight. The study divided the residence type into urban and rural. Meanwhile, maternal ages comprise < 20, 20–24, 25–29, 30–34, 35–39, 40–44, and > 44. The maternal education comprises four levels: no formal, primary, secondary, or higher education. Maternal marital status includes being married/living with a partner and divorced/widowed.

Prenatal classes offer information on pregnancy, labor, and the first few years of parenthood to expectant parents. These programs provide guidance and support. Prenatal courses may cover various topics, but they frequently cover postpartum rehabilitation, breastfeeding, alternative birth methods, and pain management to assist parents in preparing for the physical, emotional, and psychological changes that come with having a child. Prenatal education was typically provided by medical professionals like obstetricians, midwives, nurses, or trained childbirth educators [25]. The study split prenatal classes into No and Yes.

The wealth quintile of a household’s possessions was utilized in the study to determine its level of wealth. The poll graded them considering the number and variety of objects in each family’s home. The survey assessed the house’s features and a person’s extensive collection of items, including bicycles, vehicles, and televisions. The primary floor building materials, drinking water sources, and restroom amenities were all considered in the survey. The study’s scoring system was based on principal component analysis. The national wealth quintiles, broken down into the same five categories and representing 20% of the population, were determined using household scores for each participant in the pool. The poll used five categories to categorize wealth: poorest, poorer, middle, richer, and richest [26].

The study divided infants’ ages into 0–1 month, 2–3 months, and 4–5 months. The sex of the infant was boy and girl. The study divided birth weight into three groups: low birth weight (< 2,500 g, average (2,500-4,000 g), and macrosomia (> 4,000 g) [27].

### Data analysis

We applied the Chi-Square test throughout the initial research. The study used the co-linearity test to confirm no meaningful correlation between the independent variables. Then, the analysis performed a binary logistic regression test (enter method) in the final stage. We assessed the statistical significance using a 0.05 *p*-value and a 95% confidence interval (CI). To compute the statistical data for the analysis, we used IBM SPSS Statistics 26. Furthermore, we used a scatter plot to analyze the relationship between EIBF and EBF by aggregate in the province.

### Ethical approval

The national ethics committee approved the study 2021 Indonesian National Nutritional Status Survey an ethical license (LB.02.01/2/KE.248/2021). The survey used written informed consent, and respondents provided written informed consent to account for the voluntary and confidential components of the data-gathering approach.

## Results

**Fig. 1:**
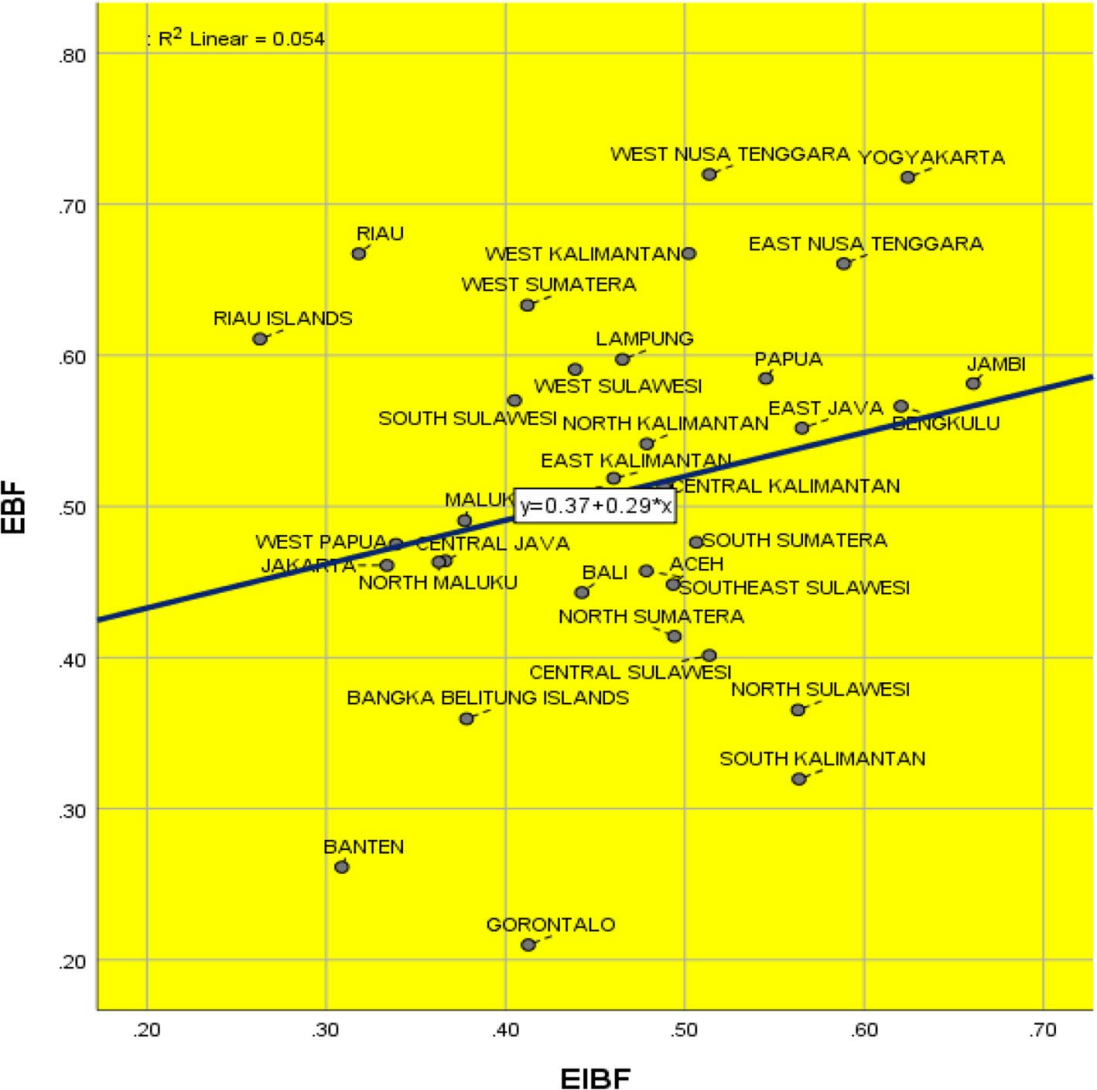
Scatter plot of the relationship between EBF and EIBF among Indonesian working mothers in 2021. Source: Visualisation by the author based on the 2021 Indonesian National Nutritional Status Survey

The result indicates that the prevalence of EBF among Indonesian working mothers in 2021 is 51.90% (95% CI 51.38%-52.48%); meanwhile, the prevalence of EIBF is 47.50% (95% CI 47.03%-47.98). Moreover, Fig. 1 displays the scatter plot of the relationship between EBF and EIBF among Indonesian working mothers. The figure indicates a positive relationship between EIBF and EBF by province among Indonesian working mothers in 2021. The higher the prevalence of EIBF, the higher the prevalence of EBF.

Table 1 shows the descriptive statistics of EIBF in Indonesia. Based on EBF, the proportion of Indonesian working mothers with EBF dominates the group performing EIBF. Regarding the residence type, Indonesian working mothers in urban areas dominate the group that achieves EIBF.

**Table 1:** Descriptive statistics of EIBF among Indonesian working mothers (*n* = 4,003)

Variable	Early Initiation of Breastfeeding		*P*-value
	No (*n* = 1,937)	Yes (*n* = 2,066)	
EBF			< 0.001
• No	56.6%	38.8%	
• Yes	43.4%	61.2%	
Residence type			< 0.001
• Urban	54.6%	53.4%	
• Rural	45.4%	46.6%	
Maternal age (in years)			< 0.001
• <20	1.5%	2.0%	
• 20–24	14.1%	14.3%	
• 25–29	28.7%	29.9%	
• 30–34	29.8%	29.2%	
• 35–39	19.4%	16.7%	
• 40–44	5.6%	6.1%	
• >44	0.8%	1.8%	
Maternal Marital Status			< 0.001
• Married/Living with Partner	99.4%	99.3%	
• Divorced/Widowed	0.6%	0.7%	
Maternal Education			< 0.001
• No formal education	1.8%	1.2%	
• Primary	37.1%	35.6%	
• Secondary	33.0%	32.1%	
• Higher	28.0%	31.2%	
Prenatal classes			< 0.001
• No	82.2%	79.3%	
• Yes	17.8%	20.7%	
Wealth status			< 0.001
• Poorest	14.7%	14.4%	
• Poorer	14.1%	15.2%	
• Middle	17.6%	16.8%	
• Richer	23.0%	24.5%	
• Richest	30.6%	29.1%	
Age of Infant (in months)			< 0.001
• 0–1	23.2%	22.7%	
• 2–3	34.9%	36.5%	
• 4–5	41.8%	40.9%	
Sex of Infant			< 0.001
• Boy	49.5%	48.2%	
• Girl	50.5%	51.8%	
Birth Weight			< 0.001
• Low birth weight (< 2500 g)	8.1%	4.4%	
• Normal (2,500-4,000 g)	83.4%	91.0%	
• Macrosomia (> 4,000 g)	8.5%	4.6%	

According to maternal age, Table 1 shows that 25–29 dominated the group of EIBF. Regarding maternal marital status, married mothers dominated all EIBF groups. Based on maternal education, Indonesian working mothers with primary education lead the group that achieves EIBF. Meanwhile, based on prenatal classes, mothers without prenatal classes dominated the group that performed EIBF. Furthermore, regarding wealth status, the most prosperous mothers lead the group that performs EIBF.

Table 1 indicates that, according to the infant’s age, 4–5 months lead the kinds that achieve EIBF. Regarding the sex of the infant, the girls dominated the group that performed EIBF. Moreover, based on birth weight, the average weight dominated all groups of EBF performance.

The following investigation was a co-linearity test. The findings demonstrate that all variables’ variance inflation factor (VIF) values are simultaneously greater than 10.00 and that the tolerance values for all variables are, on average, more significant than 0.10. After using co-linearity tests to rule out collinearity between the independent variables, the study indicated no signs of a strong relationship between two or more independent variables in the regression model.

Table 2 displays the binary logistic regression results. Based on EIBF, Indonesian working mothers with EIBF are 2.053 times more likely than those without to perform EBF (AOR 2.053; 95% CI 2.028–2.077). Moreover, the study also found all control variables related to EBF among Indonesian working mothers.

**Table 2:** The results of binary logistic regression of EBF among Indonesian working mothers (*n* = 4,003)

Predictors	EBF			
	*P*-value	AOR	95% CI	
			Lower Bound	Upper Bound
Early initiation of breastfeeding: No (ref.)	-	-	-	-
Early initiation of breastfeeding: Yes	*<0.001	2.053	2.028	2.077
Residence: Urban (ref.)	-	-	-	-
Residence: Rural	*<0.001	1.385	1.367	1.403
Maternal age: <20 (ref.)	-	-	-	-
Maternal age: 20–24	*<0.001	0.853	0.813	0.894
Maternal age: 25–29	*<0.001	1.296	1.238	1.358
Maternal age: 30–34	*<0.001	1.280	1.222	1.341
Maternal age: 35–39	*<0.001	0.906	0.864	0.949
Maternal age: 40–44	*<0.001	1.272	1.208	1.338
Maternal age: >44	**0.002	1.114	1.039	1.195
Maternal Marital: Married	. *<0.001	1.945	1.801	2.100
Maternal Marital: Divorced/Widowed (ref.)	-	-	-	-
Maternal Education: No formal education (ref.)	-	-	-	-
Maternal Education: Primary	*<0.001	0.553	0.524	0.583
Maternal Education: Secondary	*<0.001	0.364	0.344	0.384
Maternal Education: Higher	*<0.001	0.510	0.482	0.539
Prenatal classes: No (ref.)	-	-	-	-
Prenatal classes: Yes	*<0.001	1.091	1.075	1.108
Wealth: Poorest (ref.)	-	-	-	-
Wealth: Poorer	*<0.001	0.788	0.770	0.806
Wealth: Middle	*<0.001	0.648	0.634	0.663
Wealth: Richer	*<0.001	0.668	0.654	0.683
Wealth: Richest	*<0.001	0.772	0.754	0.790
Age of Infant: 0–1 month	*<0.001	2.343	2.307	2.381
Age of Infant: 2–3 months	*<0.001	1.561	1.540	1.583
Age of Infant: 4–5 months (ref.)	-	-	-	-
Sex of Infants: Boy (ref.)	-	-	-	-
Sex of Infants: Girl	*<0.001	1.275	1.260	1.290
Birth weight: Low birth weight (ref.)	-	-	-	-
Birth weight: Normal	*<0.001	1.732	1.689	1.776
Birth weight: Macrosomia	*<0.001	1.136	1.098	1.175

*AOR* Adjusted Odds Ratio, *CI* Confidence Interval

*P < 0.001; **P < 0.01

Regarding the type of residence, Table 2 shows that Indonesian working mothers in rural areas are 1.385 times more likely than those in urban areas to perform EBF (AOR 1.385; 95% CI 1.367–1.403). Moreover, the result indicates maternal age is associated with EBF performance among Indonesian working mothers.

Table 2 shows that married mothers are 1.945 times more likely to achieve EBF than divorced/widowed (AOR 1.945; 95% CI 1.801-2.100). According to education level, Indonesian working mothers with all education levels are less likely than those without formal education to achieve EBF. Meanwhile, based on prenatal classes, mothers with prenatal classes are 1.091 times more likely to perform EBF than those without (AOR 1.091; 95% CI 1.075–1.108). Furthermore, based on wealth status, Indonesian working mothers in all groups are less likely than the poorest to achieve EBF.

According to the infant’s age, Table 2 indicates that infants of all ages are more likely than 4–5 months to achieve EBF. On the other hand, infant girls are 1.275 times more likely to perform EBF than infant boys (AOR 1.275; 95% CI 1.260–1.290). Moreover, based on birth weight, infants of all weights are more likely than those of low birth weight to achieve EBF.

## Discussion

The result indicated that EIBF was related to EBF achievement among Indonesian working mothers. Working mothers who had dominant EIBF, 61.2%, gave EBF. According to WHO standards, this proportion is included in the excellent category [28]. Other factors related to EBF in working women in Indonesia are the age of marriage, the status of living with a partner, the mother’s education, wealth status, the baby’s age, the baby’s sex, and the baby’s weight. Research that is in line with this research is research in Ethiopia, which shows that EIBF is dominantly related to the implementation of EBF administration [29]. Another study in China reported that EIBF is related to EBF in formally working mothers aged six months and older on paid maternity leave [30].

Similarly, studies in Northern Uganda and similar studies show that EIBF is a significant factor associated with EBF [29, 31]. The EIBF in Ethiopia demonstrated that a factor associated with premature cessation of EBF was that breastfeeding was not initiated within the first hour of birth. Studies at Columbia show that the prevalence of EIBF within the first hour after birth is 66%. Mothers who deliver by cesarean section do not perform EIBF compared to mothers who give birth vaginally. There is a significant correlation between cesarean section and the risk of delaying EIBF [32]. A study in Milan, Lombardy, Italy, showed that difficulty giving EBF was a failure of EIBF due to difficulty in breastfeeding, cracked nipples, perception of insufficient milk supply, pain, and fatigue. Mothers’ perception of inadequate breast milk, failure to thrive, mastitis, and returning to work is related to the risk of not being given EBF [33].

This study found that Indonesian working mothers with EIBF were twice as likely to have EBF as those who were not working. Mothers who do EIBF are more likely to do EBF. Giving EBF to working mothers in Indonesia is related to other variables, namely mothers who work in rural areas, mother’s age, married mothers, all levels of primary education, mothers with classes of pregnant women, wealth status, baby’s age, and baby’s weight. Studies of interrupted skin-to-skin contact after birth are consistent with this research, increasing the likelihood of a negative breastfeeding experience one week after delivery. It is essential to support factors that can enhance a successful early breastfeeding experience [34, 35].

In contrast, this retrospective observational cohort study in Valencia (Spain) demonstrated no significant association between EIBF in the first two hours after birth and EBF at six months postpartum. However, there was a clear and significant correlation between breastfeeding efficiency as assessed by how well the baby latches on, swallow sounds, nipple type, comfort, and holding position, as well as maintenance of EBF at six months. Low breastfeeding efficiency is associated with discontinuation of breastfeeding within six months. Not only is EIBF prioritized, but it is also crucial that health interventions can increase information about breastfeeding efficiency and empower mothers to increase the success of EBF, which is proven beneficial for the health of babies and mothers [36].

WHO recommends EBF for the first six months of life, followed by continued breastfeeding with suitable complementary foods for up to 2 years or beyond as one of the global nutrition targets in 2025. WHO and UNICEF call on manufacturers of breastmilk substitutes to fully commit to compliance with the Code of International Marketing of Breast Milk Substitutes [28]. The Global Breastfeeding Collective has identified seven policy priorities for countries to protect, promote, and support breastfeeding. The Nutrition for Growth Summit 2021 announced several firm commitments from governments, development partners, and civil society partners to improve nutrition, mainly through increased investment in EBF [37]. Indonesia is one of the countries that determine EBF policies. The EBF policy in working areas is contained in the government regulations of the Republic of Indonesia. Managers of workplaces and organizers of public facilities are required to provide special facilities for breastfeeding and expressing breast milk according to the company’s capabilities. Organizers of public health service facilities must support the success of the EBF program by putting together ten steps to successful breastfeeding. Workplace managers are required to provide opportunities for working mothers to breastfeed their babies exclusively or express breast milk during working hours at the workplace [38].

Based on the type of residence, working mothers in Indonesia who work in rural areas are more likely to experience EBF than mothers in urban areas. The condition is consistent with research showing that agricultural employment is positively related to EIBF and EBF. Industry and business-related jobs are negatively associated with EBF. Occupational factors influencing breastfeeding implementation are job benefits, travel time, work environment, and labor intensity [39, 40]. Research in Ghana shows that the main factors influencing working mothers in EIBF related to the workplace are the length of maternity leave, closing time, absence of maternity policies in the organization, inadequate institutional support, and work-family balance [39].

This study found several maternal demographics related to EBF performance among working mothers in Indonesia: age, marriage, and education. The study results indicate that EIBF is dominant in mothers aged 25–29. Consistent with these results, the average age of working mothers who apply for EBF is 27.44 years, and the majority are married [41, 42]. EBF practice is dominant at 20–29 years [43, 44]. Giving EBF to working mothers is supported by marital status. Married working mothers are likelier to achieve EBF than widows/divorced [19, 45]. By level of education, working mothers in Indonesia with all levels of education are less likely to achieve EBF than mothers without formal education. The results of this study are exciting; EBF in working mothers is supported by mothers who do not have formal education. Working mothers who have practiced EBF have various obstacles [46]. The main reasons for not doing EBF are work pressures and medical conditions. Ninety-nine percent of mothers do not have workplace facilities that support breastfeeding (such as a nursing room, nursery, refrigerator, and privacy) [47].

The study results prove that working mothers without education prefer breastfeeding their babies exclusively. Working mothers with formal education are likelier to find work in the proper and professional sectors. Early return to work, insufficient support for breastfeeding, low milk, and lack of time are the main barriers to working mothers for EBF. Lack of breastfeeding breaks, lactation sites, and milk storage facilities in primary health care are the main work-related barriers to EBF continuity [48, 49]. Consistent with the study in Yaoundé, it also supports the mother’s higher education as a risk factor for non-adherence to EBF and continued breastfeeding at two years [19, 50]. There are four reasons for occupational factors that influence breastfeeding practices: 1) employment benefits, 2) travel time, 3) work environment, and 4) labor intensity [51].

Meanwhile, based on the class of pregnant women, mothers who attended classes for pregnant women were more likely to do EBF than those who did not. The results prove that courses for pregnant women positively impact the practice of giving EBF to working mothers. Mothers who work and receive education about breastfeeding are also associated with 12 months of continued breastfeeding [52]. These findings are consistent with postnatal classes. Mothers who do not receive breastfeeding counseling after giving birth are less likely to practice EBF than those who receive services [53]. These findings support evidence that members of a breastfeeding support group have a higher chance of EBF compared to those who are not members of a breastfeeding support group.

Based on wealth status, Indonesian working mothers across all groups are less likely than the poorest to achieve EBF. The wealthier the working mothers are, the more work is to be done. The condition may hinder the medium wealth index from practicing less EBF than mothers with a low wealth index [54]. Mothers with the poorest wealth index have the highest likelihood of EBF than wealth index groups [55]. Mothers who work as homemakers provide more EBF for their babies than working mothers [56]. This empirical evidence suggests wealth status is not always inversely related to EBF practices.

In addition, this study also found three baby characteristics associated with EBF performance: age, gender, and birth weight. Babies of all ages are likelier to achieve EBF at 4–5 months. In contrast, a study in Ethiopia stated that infants aged 4–5 months were less likely to get EBF [57]. On the other hand, baby girls are more likely to have EBF than baby boys. The situation is consistent with a study in Ethiopia proving that baby girls are individual-level determinants significantly associated with EBF [57]. Based on birth weight, babies of any weight are more likely to achieve EBF than those of low birth weight. However, in contrast to the study, children with standard birth weights have a smaller chance of breastfeeding (OR = 0.87) than children with low birth weights [58]. This empirical evidence shows that the characteristics of the baby can be a support or a barrier to EBF.

### Strength and limitation

The study needs to analyze a large amount of data to provide conclusions at the national level. The 2021 Indonesian National Nutritional Status Survey served as a secondary data source for this study, and we solely looked at the survey’s provided variables. The study’s conclusions exclude several other elements connected to EBF in past studies, including birth order, the number of children born, their value, whether they had a fever or diarrhea, and the total number of children born [23, 59, 60].

## Conclusions

Based on the study’s results, the study concluded that EIBF was related to EBF. Indonesian working mothers with EIBF were two times more likely than those without to perform EBF. Regarding the study’s results, to increase EBF coverage, the government needs to release policies that strengthen the occurrence of EIBF among working mothers. One policy that needs to be considered is giving husbands leave so they can accompany their wives during birth and support their EIBF performance at the health facility.

This study was carried out using a quantitative approach with study results that tend to be superficial. Further studies are still needed with a qualitative approach to dig deeper into several situations that can influence EBF practices among working mothers in Indonesia, including workplace barriers, cultural norms, and economic factors.

## Data Availability

The author cannot publicly disclose the data since neither a third party nor the Ministry of Health of the Republic of Indonesia, the data’s owner, is authorized to do so. For researchers who meet the requirements for access to sensitive data, the 2021 Indonesian National Nutrition Status Survey data set is accessible online at https://layanandata.kemkes.go.id/.
